# Rhombencephalitis With Long Segment Transverse Myelitis: A Presentation With a Rare Etiology

**DOI:** 10.7759/cureus.69386

**Published:** 2024-09-14

**Authors:** Prashant Dubey, Seema Seth, Prajwal Rao, Pravin Naphade

**Affiliations:** 1 Department of Internal Medicine, Rohilkhand Medical College and Hospital, Bareilly, IND; 2 Department of Neurology, Dr. D. Y. Patil Medical College, Hospital and Research Centre, Dr. D. Y. Patil Vidyapeeth (Deemed to be University), Pune, IND

**Keywords:** encephalitis, herpes, long segment transverse myelitis, meningitis, rhombencephalitis

## Abstract

Rhombencephalitis is an inflammatory disease affecting the hindbrain (brainstem and cerebellum). The causes of rhombencephalitis can be divided into infections, autoimmune conditions, and paraneoplastic syndrome. Early onset rhombencephalitis is associated with demyelinating disorders or Epstein-Barr virus infection. Infections like Listeria and paraneoplastic disorders were seen in older individuals. Transverse myelitis includes pathobiological heterogeneous syndrome characterized by acute or subacute spinal cord dysfunction resulting in paraparesis, a sensory level, and autonomic (bladder, bowel, and sexual) impairment below the level. A lesion of the spinal cord where three or more vertebral segments are involved is called longitudinally extensive transverse myelitis (LETM). One of the most distinct causes of LETM is neuromyelitis optica spectrum disorder.

Here, we describe a 34-year-old male who presented with quadriparesis, multiple cranial nerve palsies with sensory, bowel, and bladder involvement, along with altered sensorium with a preceding history of fever five days back. His magnetic resonance imaging (MRI) of the brain and spine was suggestive of rhombencephalitis with long-segment transverse myelitis. He later developed an intracranial hemorrhage during the hospital stay. His cerebrospinal fluid BioFire (BioFire Diagnostics, LCC, Salt Lake City, UT) was positive for herpes simplex virus 1.

## Introduction

Rhombencephalitis is an inflammatory condition that targets the hindbrain, encompassing the brain stem and cerebellum. This disease presents a wide array of etiology, some even life-threatening if not promptly and correctly treated [1]. Rhombencephalitis arises from various causes, categorized into infectious diseases (Listeria, enterovirus 71, and herpes virus), autoimmune disorders (predominantly Behcet's disease), and paraneoplastic syndromes. The latter include antibodies like anti-Mo, anti-Tr, anti-Hu, and anti-Ma, commonly associated with small-cell lung carcinoma [2].

Longitudinally extensive transverse myelitis (LETM) is an inflammatory condition affecting the spinal cord, typically spanning three or more contiguous segments [3]. Its clinical presentation involves single or multiple episodes characterized by weakness in the legs (paraparesis), arms and legs (tetraparesis or quadriparesis), sensory deficits, and potential bowel/bladder dysfunction, sometimes extending to respiratory complications. Etiologies of LETM include neuromyelitis optica (NMO), various infectious agents, autoimmune disorders such as systemic lupus erythematosus and sarcoidosis, as well as arteriovenous fistulas [4].

Here, we present the case of a 34-year-old male who exhibited quadriparesis, multiple cranial nerve palsies, and altered mental status with a history of fever five days before presentation. MRI findings indicated rhombencephalitis accompanied by long-segment transverse myelitis. Further testing of cerebrospinal fluid (CSF) using BioFire (BioFire Diagnostics, LCC, Salt Lake City, UT) confirmed positivity for herpes simplex virus 1 (HSV-1).

## Case presentation

A 34-year-old male without any preexisting medical conditions presented with an acute onset of high-grade fever associated with generalized body pain lasting for two days. Although the fever subsided with medication over the next three to four days, he continued to experience persistent generalized body pain. He developed gradually worsening weakness, starting in his lower limbs and progressing to involve his upper limbs, rendering him unable to walk. He also experienced abdominal distension and retention of stool and urine simultaneously. Over the next few days, the patient experienced progressive difficulty swallowing, leading to nasal regurgitation of food. His speech became incomprehensible, followed by a decline in consciousness. Upon arrival at the hospital, he was drowsy but responsive to simple verbal commands. His vital signs were unstable, marked by rapid blood pressure and pulse rate fluctuations. The neurological assessment revealed the involvement of multiple cranial nerves (bilateral fourth, sixth, seventh, ninth, and tenth cranial nerves). He exhibited generalized muscle weakness, hypotonia, and a complete absence of muscle strength (0/5) in both upper and lower limbs. Sensory examination was challenging but indicated impaired pinprick sensation extending up to the upper part of the face. Deep tendon reflexes were absent bilaterally, and the plantar reflexes were bilateral extensor response.

**Figure 1:**
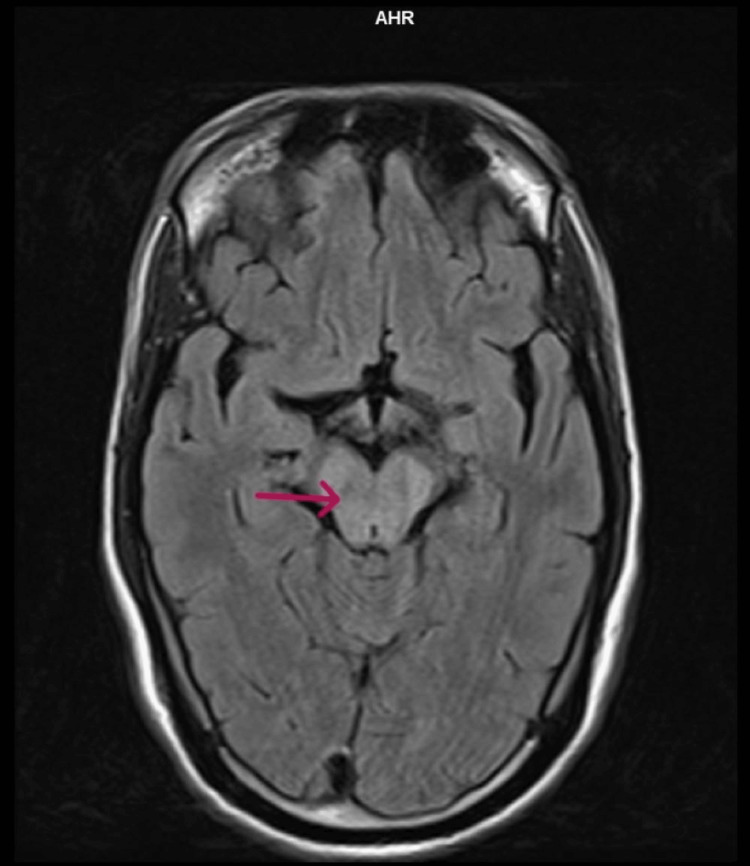
T2 FLAIR hyperintensities involving midbrain (arrow) on the axial section FLAIR: fluid-attenuated inversion recovery

**Figure 2:**
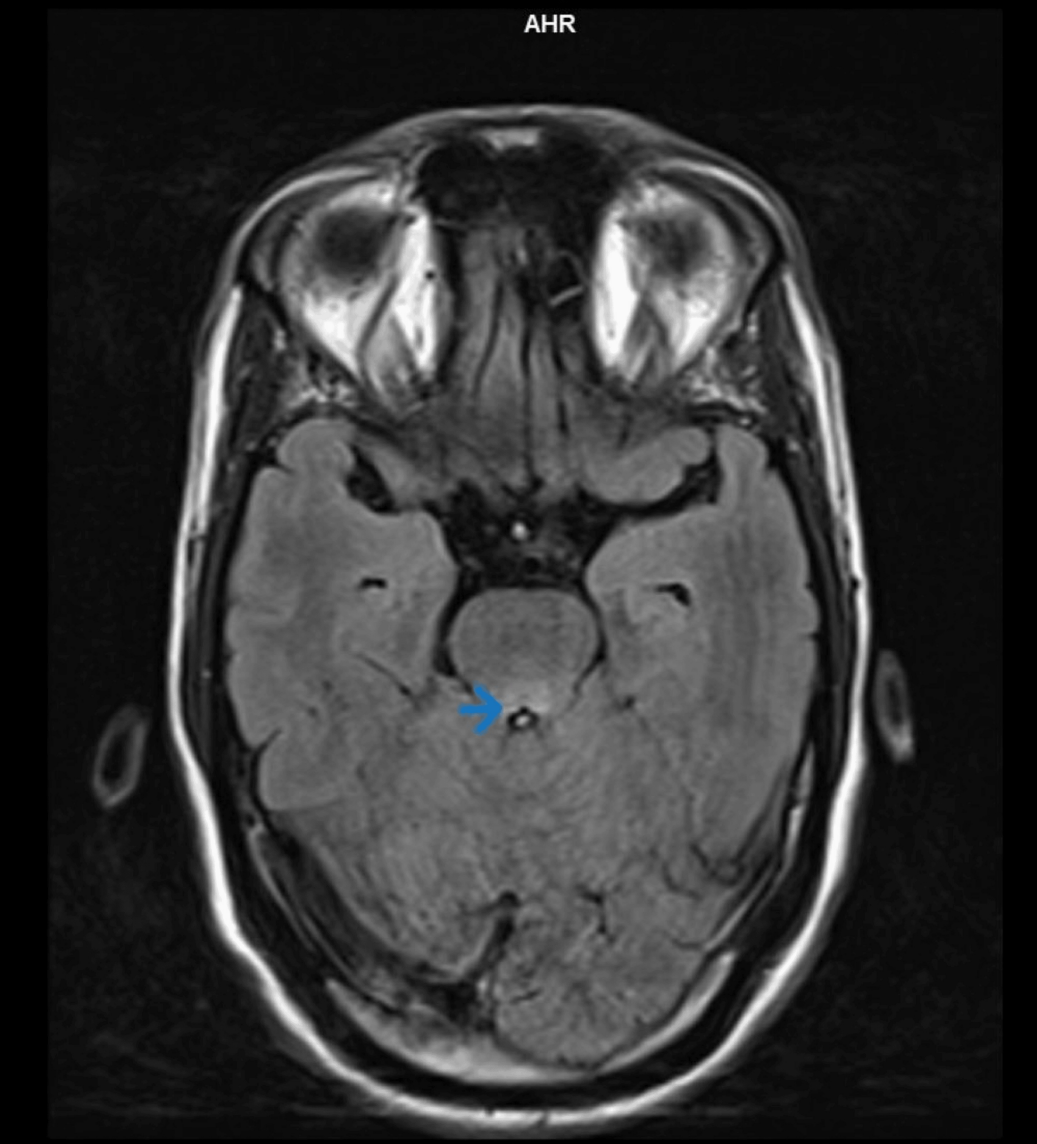
T2 FLAIR hyperintensities involving dorsal pons (arrow) on the axial section at the level of mid-pons FLAIR: fluid-attenuated inversion recovery

**Figure 3:**
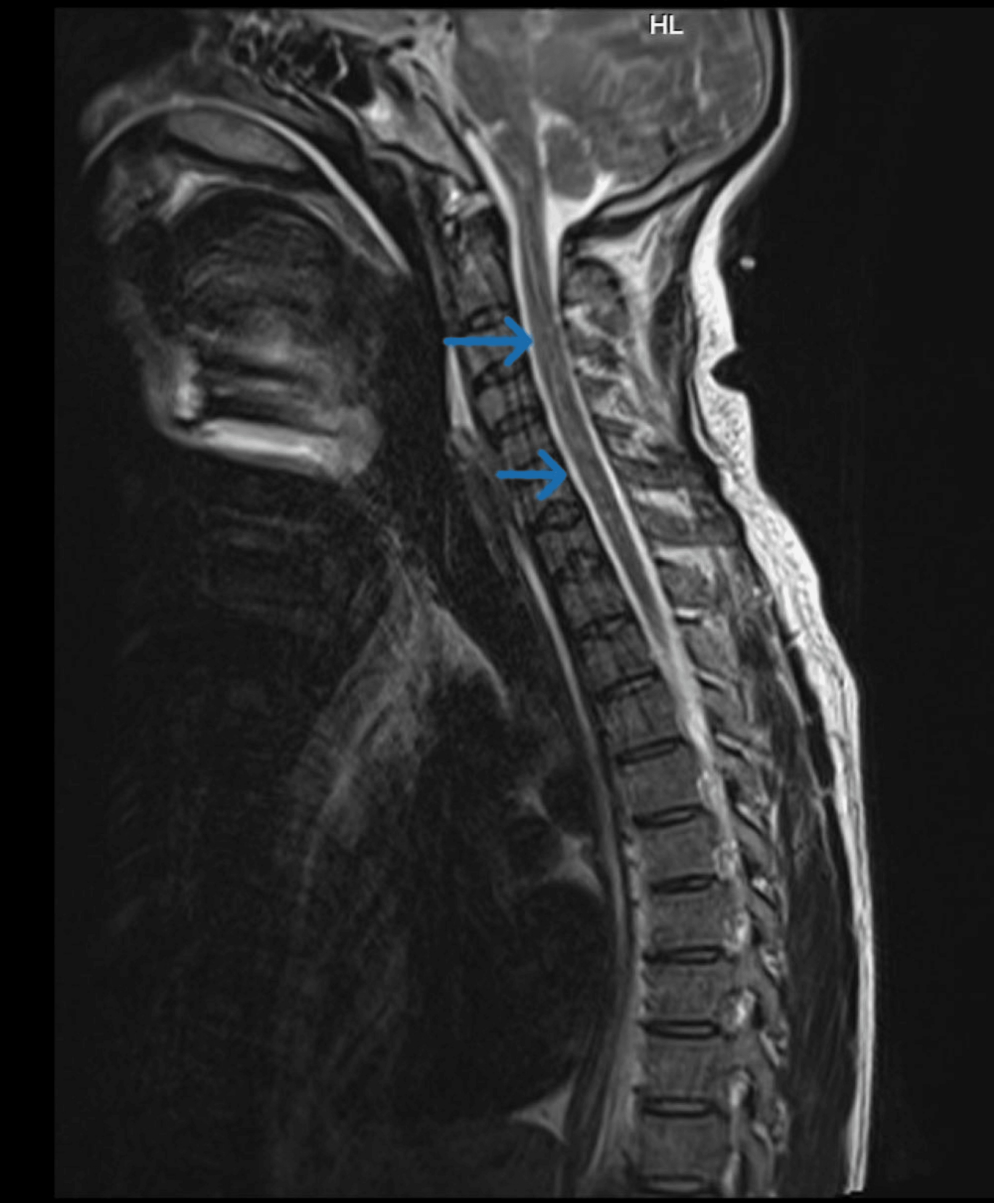
T2 hyperintense signals in the cervicodorsal spine (arrows) extending from brain stem on the sagittal section

**Figure 4:**
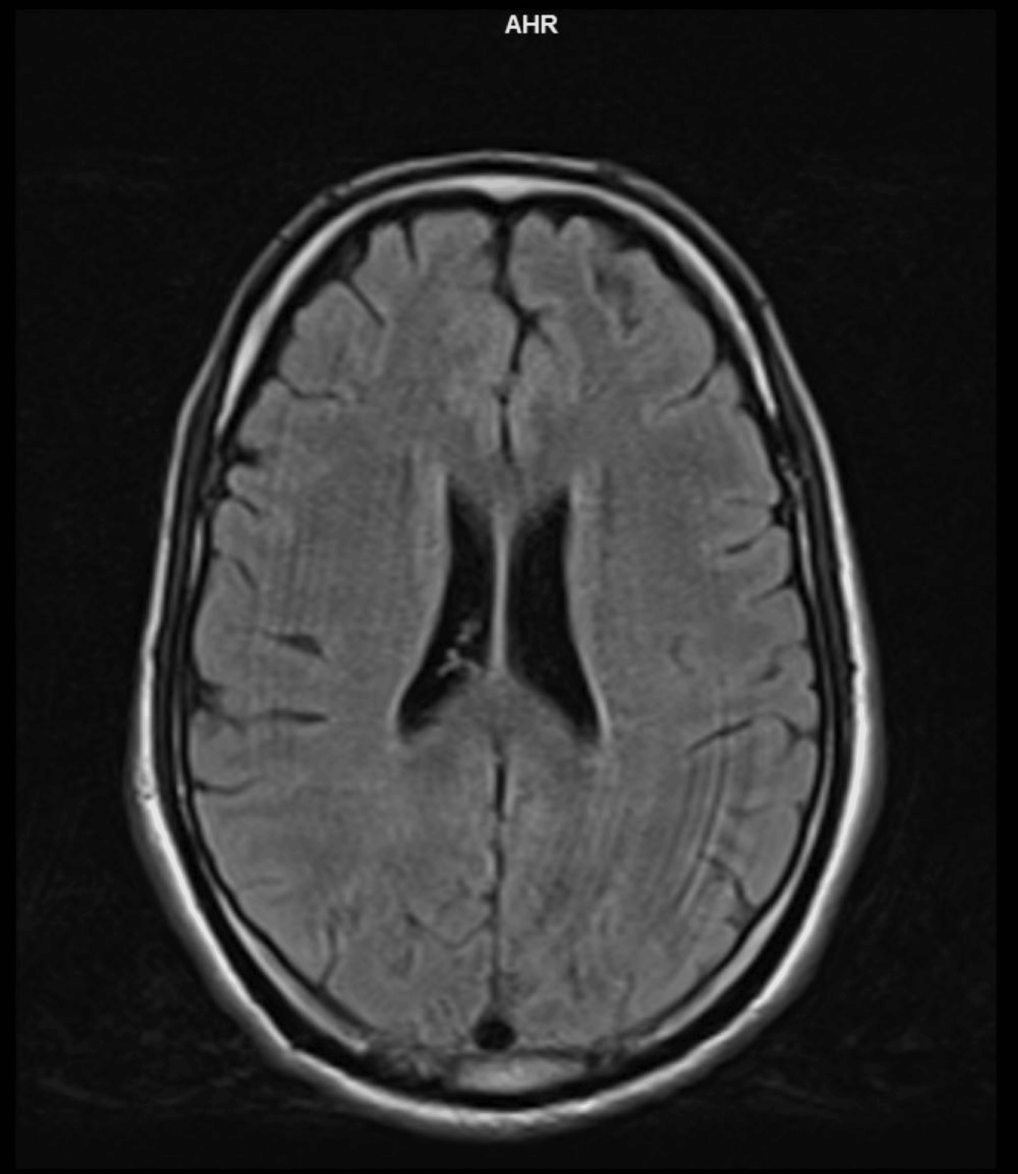
T2 FLAIR axial section showing no involvement of the cortex FLAIR: fluid-attenuated inversion recovery

**Figure 5:**
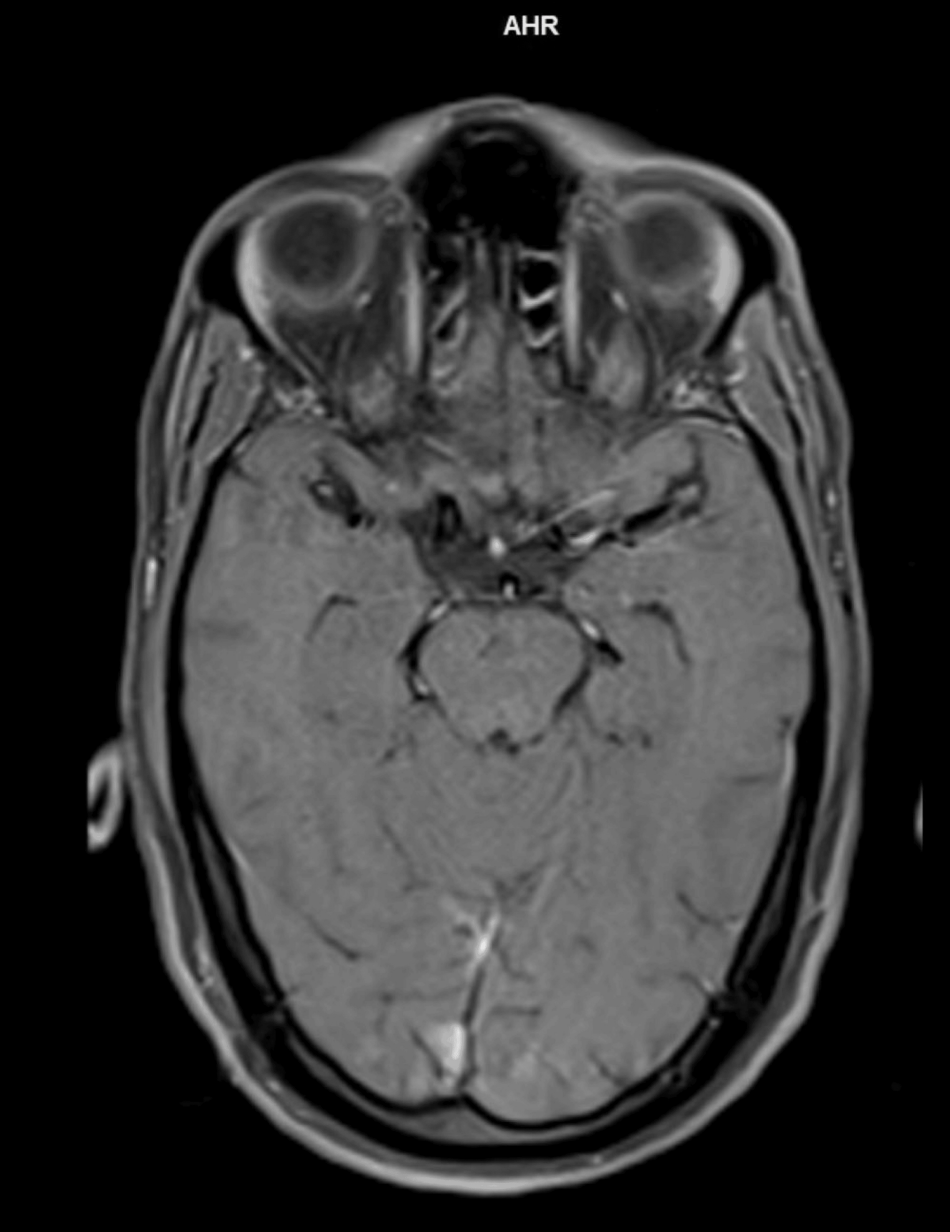
T1 axial postcontrast image at the level of pons showing no contrast uptake

Based on the clinical history and examination findings, a provisional diagnosis of brain stem encephalitis with long-segment transverse myelitis was considered. The patient underwent appropriate investigations, including an MRI of the brain with whole spine screening using contrast. The imaging revealed T2 and fluid-attenuated inversion recovery hyperintensities in the midbrain (Figure 1), dorsal pons, and medulla (Figure 2), with extension into the cervical and dorsal spinal cord (Figure 3). Importantly, no cortex involvement was observed (Figure 4), and no postcontrast enhancement was observed in any lesions (Figure 5).

**Figure 6:**
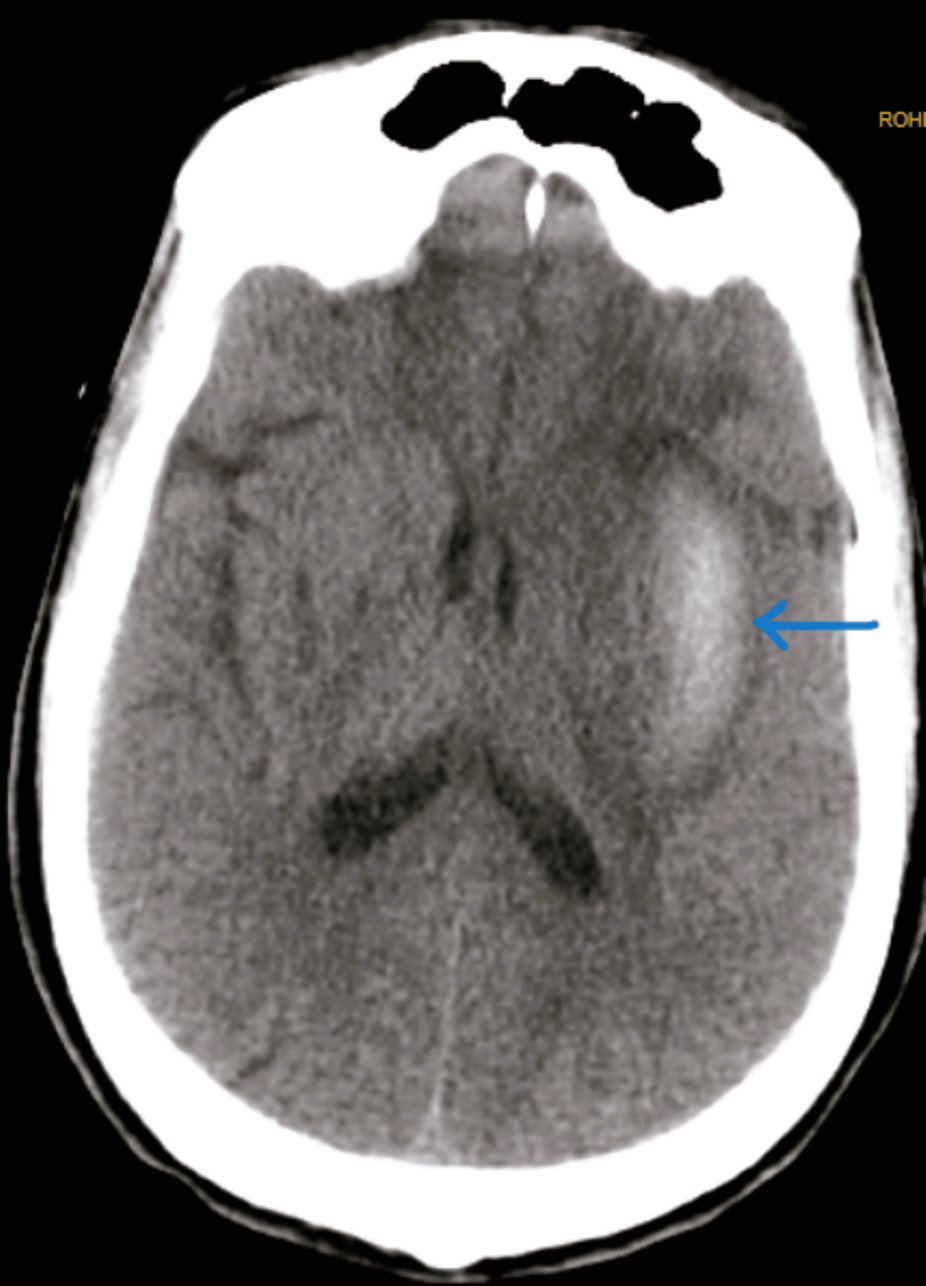
CT brain axial section showing an intraparenchymal hemorrhage (arrow) in the left external capsule

The patient was started on intravenous immunoglobulins and methylprednisolone 1 gm, given for five days along with antibiotics and acyclovir. Serum NMO, myelin oligodendrocyte glycoprotein, CSF oligoclonal band, antinuclear antibody, and antineutrophil cytoplasmic antibody were all negative. Based on the cerebrospinal fluid (CSF) examination, the total leucocyte count is 44, the protein level is 62, and the differential leucocyte count shows 10% neutrophils and 90% lymphocytes. The sugar level is 88 mg/dL (corresponding blood sugar: 108 mg/dL). Looking at multiple cranial nerve palsy, a ganglioside panel was sent, which was negative. CSF BioFire was sent. CSF for West Nile virus and Japanese encephalitis were also sent. The patient did not show any improvement despite two cycles of intravenous immunoglobulins and steroid therapy. The patient was on ventilatory support during his illness. Initially, he was maintained on synchronized intermittent mandatory ventilation mode of the ventilator and had a Glasgow Coma Scale (GCS) of E2VTM3, but on day 13 of admission, he became very drowsy, and GCS fell to E1VTM1. CT of the brain was done, and there was a suggestive hemorrhage in the left external capsule (Figure 6).

Meanwhile, his CSF BioFire report came out to be positive for HSV-1. The patient was already receiving treatment with antiviral, antibiotics, and steroids. Additionally, antiedema drugs such as mannitol and acetazolamide were included in the treatment regimen. However, there was not much improvement in his condition. He developed ventilator-associated pneumonia and later died of ICU-related complications.

## Discussion

Rhombencephalitis has a varied range of etiologies, some of which are life-threatening. Patients with rhombencephalitis presenting at an early age in previous studies had demyelinating disorders or Epstein-Barr virus (EBV) infection as the main etiology. Previous studies have shown Listeria infection and paraneoplastic syndrome occurring generally in older individuals [5]. Ataxia was a common presentation in diseases such as subacute cerebellar degeneration and EBV encephalitis. Cranial nerve involvement correlated well with infectious causes [6]. Fever and altered sensorium were associated with infectious causes like Listeria [7]. Long-segment transverse myelitis (LETM) occurs in all age groups. Various studies have shown a slight male predominance [8]. Clinical features depend on the type of myelitis, partial or complete. It also depends on the segment of the cord involved. Neuromyelitis optica spectrum disorder has been associated with recurrence, involvement of cervical cord, and associated optic neuritis [9].

Herpes simplex encephalitis (HSE) is primarily caused by HSV-1, with herpes simplex virus 2 (HSV-2) being responsible for a small minority of cases, typically less than 10%. This condition is characterized by acute or subacute onset and involves significant cerebral dysfunction due to the viral infection affecting the brain. The temporal lobes and adjacent limbic systems are commonly affected in HSE, leading to symptoms such as altered mental status, focal neurological deficits, and, sometimes, seizures. This temporal lobe involvement preference is a hallmark feature of HSE [10]. In newborns, the pattern of brain involvement may differ compared to older children and adults. Instead of focal lesions, newborns often exhibit more diffuse brain involvement, which can lead to a broader range of neurological symptoms and complications. Meningeal congestion is frequently observed in HSE. This inflammation of the meninges can contribute to the clinical presentation and may be evident in diagnostic imaging or during autopsy examinations. The postinfection autoimmune reactions can be a molecular mimicry between the agent causing infection, antigens of the central nervous system, and inflammatory processes secondary to microbial superantigens. Intracranial hemorrhage is an uncommon occurrence in herpes encephalitis, even when antiviral therapy is promptly initiated. Typically, the brain shows a necrotizing infiltrate with scattered small areas of hemorrhage. However, the formation of a significant hematoma is rare and tends to manifest during the second week following the initial presentation [11]. In previous studies, pathological examination of the evacuated hematoma revealed fibrinoid necrosis, indicative of small vessel vasculitis. This inflammatory process likely caused endothelial damage, leading to the observed hemorrhage.

In summary, HSV-1 is the primary causative agent of HSE in both children and adults, affecting specific brain regions like the temporal lobes and limbic system, whereas HSV-2 is less commonly associated with this condition [12]. Immunocompromised patients may have atypical and more extensive tissue involvement, with lesions in the brainstem, cerebellum, and cerebral cortex, but this is rarely seen. Rhombencephalitis as a presenting feature of HSV-1 has been less reported [13].

Our patient had the full spectrum of clinical features, including ataxia, quadriparesis, multiple cranial nerve involvement, and altered sensorium associated with fever. He had rhombencephalitis with long-segment transverse myelitis [14]. He developed intracranial hemorrhage, which is a rare complication of HSV encephalitis during illness [15].

## Conclusions

The case report brings out some important learning points. HSV-1, though rare, may cause rhombencephalitis and long-segment transverse myelitis. A rare but documented complication of HSE is intraparenchymal hemorrhage. Early antiviral therapy may be considered empirically in cases of rhombencephalitis where the cause is not evident.
